# Impact of a case-management intervention for reducing emergency attendance on primary care: randomised control trial

**DOI:** 10.3399/BJGP.2021.0545

**Published:** 2022-05-17

**Authors:** Jonathan N Cohen, An Nguyen, Meena Rafiq, Paul Taylor

**Affiliations:** Institute of Health Informatics, University College London, London.; Health Navigator Ltd.; Epidemiology of Cancer and Healthcare Outcomes, Institute of Epidemiology and Health Care, University College London, London.; Institute of Health Informatics, University College London, London.

**Keywords:** artificial intelligence, case management, frequent attenders, healthcare utilisation, high-intensity users, primary health care

## Abstract

**Background:**

The impact on primary care workload of case-management interventions to reduce emergency department (ED) attendances is unknown.

**Aim:**

To examine the impact of a telephone-based case-management intervention targeting people with high ED attendance on primary care use.

**Design and setting:**

A single-site data extract from a larger randomised control trial, using the patient-level data from primary care electronic health records (2015–2020), was undertaken.

**Method:**

A total of 363 patients at high risk of ED usage were randomised to receive a 6-month case-management intervention (253 patients) or standard care (110 patients). Poisson regression models were used to calculate monthly rates of primary care use over time for the 2 years post-randomisation, comparing both arms. Usage was subclassified into face-to-face, telephone, letter, and community and secondary care referrals, stratified by patient demographics.

**Results:**

No significant difference was found in the mean annual rate of primary care events between the intervention and control arms (*P* = 0.70). Secondary care referrals saw a 26% reduction in the mean annual referral rate (incident rate ratio [IRR] 0.74, 95% confidence interval [CI] = 0.64 to 0.86, *P*<0.001) and letters sent increased by 6% in the intervention arm compared with the control arm (IRR 1.06, 95% CI = 1.01 to 1.11, *P* = 0.01). In the case-managed arm, in patients aged ≥80 years there was a 33% increase in primary care usage (IRR 1.33, 95% CI = 1.28 to 1.40, *P*<0.001); with a corresponding 10% decrease in patients aged <80 years when compared with controls (IRR 0.90, 95% CI = 0.87 to 0.92, *P*<0.001).

**Conclusion:**

A targeted case-management intervention to reduce ED attendances did not increase overall primary care use. Redistribution of usage is seen among some patient groups, particularly older people, which may have important implications for primary healthcare planning.

## INTRODUCTION

Over the past decade, the number of patient visits to emergency departments (EDs) has increased across almost all high-income countries.^1^ In the UK, there was an estimated 9% increase in ED attendance between 2013 and 2017.^2^ This strain has been further highlighted over the past 18 months, with the COVID-19 pandemic causing surges in ED attendance rates and placing unprecedented demands on hospital emergency services.^3^

Many ED attendances are avoidable or would be more appropriately treated by other healthcare providers, including GP and community services. NHS England found 24% of ED attendances were inappropriate or avoidable,^2^ with 11% discharged without requiring treatment and 39% receiving advice only.^4^ Additionally, a small number of patients account for a disproportionately high number of ED attendances;^5^^–^^7^ 5% of patients are estimated to account for >25% of all ED visits.^8^

Improving access to primary care services^9^^–^^11^ and promoting continuity of care^9^^,^^12^^,^^13^ effectively reduces ED use. Additionally, patients who are confident in managing their own health conditions have 32% fewer attendances at EDs and 38% fewer emergency hospital admissions.^14^

Case-management strategies are a proposed solution to support these factors to reduce ED attendances. These are *‘a collaborative process of assessment, planning, facilitation, care coordination, evaluation and advocacy for options and services to meet an individual’s … comprehensive health needs’*.^15^ Case-management strategies have been shown to reduce unscheduled emergency care and costs by identifying patients at a higher risk of unplanned hospital attendance, often with multiple comorbidities and psychosocial factors, and coordinating patients’ care with available services.^16^^,^^17^ They can produce a 12%–26% reduction in emergency hospital admissions in people who frequently attend EDs.^18^^–^^20^ However, no evaluation has been conducted of their impact on wider healthcare service use, particularly primary care. This is important as any diversion of patient care from EDs may increase demands on primary care, itself a system under increasing strain.^21^^–^^23^

This study set out to examine the effect of a case-management intervention designed to reduce ED attendances on primary care use and to quantify in granular detail the impact on specific aspects of primary care services.

## METHOD

### Study setting

This single-site study in the Vale of York Clinical Commissioning Group is part of an ongoing larger parallel, two-arm randomised control trial (RCT) being conducted by Nuffield Trust and Health Navigator (Integrated Research Application System project ID: 173319; and clinicaltrials. gov ID: 2015–000810-23). The study is assessing the impact of a telephone-based case-management health coaching intervention using an algorithm-generated selection of patients at high risk of future ED attendance. Recruitment started in August 2015 and is ongoing.

**Table:** How this fits in

Case-management interventions, if carefully targeted at the right patient population, successfully reduce emergency department (ED) attendances. Their impact on primary care use is, however, unknown. This study showed that a case-management intervention to reduce ED attendances did not increase overall primary care usage. In addition, it led to a 26% reduction in referrals to secondary care services in patients receiving the intervention. The intervention had differing effects on primary care use in specific patient groups, with an increase in use in those aged ≥80 years and a decrease in those aged <80 years. This may represent a redistribution of services to those with greater clinical need with important associated implications for primary care service planning and provision.

### Study population

Daily patient-level data on hospital attendances were obtained from York Teaching Hospital and linked to primary care records. An artificial intelligence-driven risk-prediction algorithm identified patients at high risk of becoming heavy users of emergency and non-elective services (see Supplementary Box S1 and Supplementary Figures S1 and S2 for protocol details) who were invited to participate in the trial. Consented patients were randomised using an online random-sequence generator to either the intervention or control group using a 2:1 ratio in favour of the intervention group.

### Study intervention

Patients in the intervention group received an initial face-to-face meeting to discuss the intervention, followed by a telephone-based case-management programme with regular 15-minute telephone calls from a Health Navigator health coach over a 6-month period (see Supplementary Box S1 for protocol details). A personalised care plan was developed, and patients received motivational conversations, support for self-care, patient education, and coordination of social and medical services. Motivational conversations were informed by existing theories;^24^^,^^25^ components included demonstrating empathy, dealing with resistance, supporting self-efficacy, and developing autonomy. No medical advice or treatment was delivered. Patients in the control group received standard care, defined here as the provision of social prescribing and community services delivered by primary care and the local community trust.

### Data collection and management

Pseudo-anonymised data were extracted from GP electronic health records (EHRs) including age, sex, Index of Multiple Deprivation (IMD) (as a marker of socioeconomic status), and primary care use (rate of patient contacts) during the 6 months before and 2 years following the intervention. Patient contacts were stratified into face-to-face consultations, telephone contacts, and letters sent to patients (containing, for example, test results and appointment invitations), with the date and type of each primary care contact recorded. Data on GP referrals (date and destination) were collected as a secondary outcome measure to reflect the impact of the intervention on broader GP workload. Referrals were subclassified into community or secondary care referrals to additionally assess the impact on use of community services.

### Statistical analysis

To compare baseline characteristics between patients in the intervention and control arms, χ^2^ tests were used. Poisson regression analyses were used to calculate the annual rate of all primary care events for the intervention and the control arms by calculating the total number of events per person per year in each arm. Incident rate ratios (IRRs) were estimated to compare the rate in the two arms. To obtain a detailed understanding of the effect of the intervention on different aspects of GP workload, separate Poisson models were used to examine the rates of face-to-face consultations, telephone calls, letters, and community and secondary care referrals in both arms.

To explore if any differences in primary care use were limited to specific patient groups, the annual rate of all primary care events was estimated after stratifying by age, sex, and IMD quintile.

Further Poisson regression analyses were used to compare the rate of all primary care events over time between the two arms by estimating the monthly rates per patient for 6 months before and 24 months after the intervention. Monthly IRRs with corresponding 95% confidence intervals (CIs) were estimated to compare each arm. These analyses were repeated using separate models for face-to-face consultations, telephone calls, letters sent, community referrals, and secondary care referrals to observe how specific types of primary care use changed over time.

Further analyses were conducted stratifying the monthly rate of all primary care events over time by age and sex to see if trends were similar across these groups. All analyses were conducted according to intention-to-treat principles and using Stata (version 15).

## RESULTS

### Descriptive analysis

**Figure 1.**
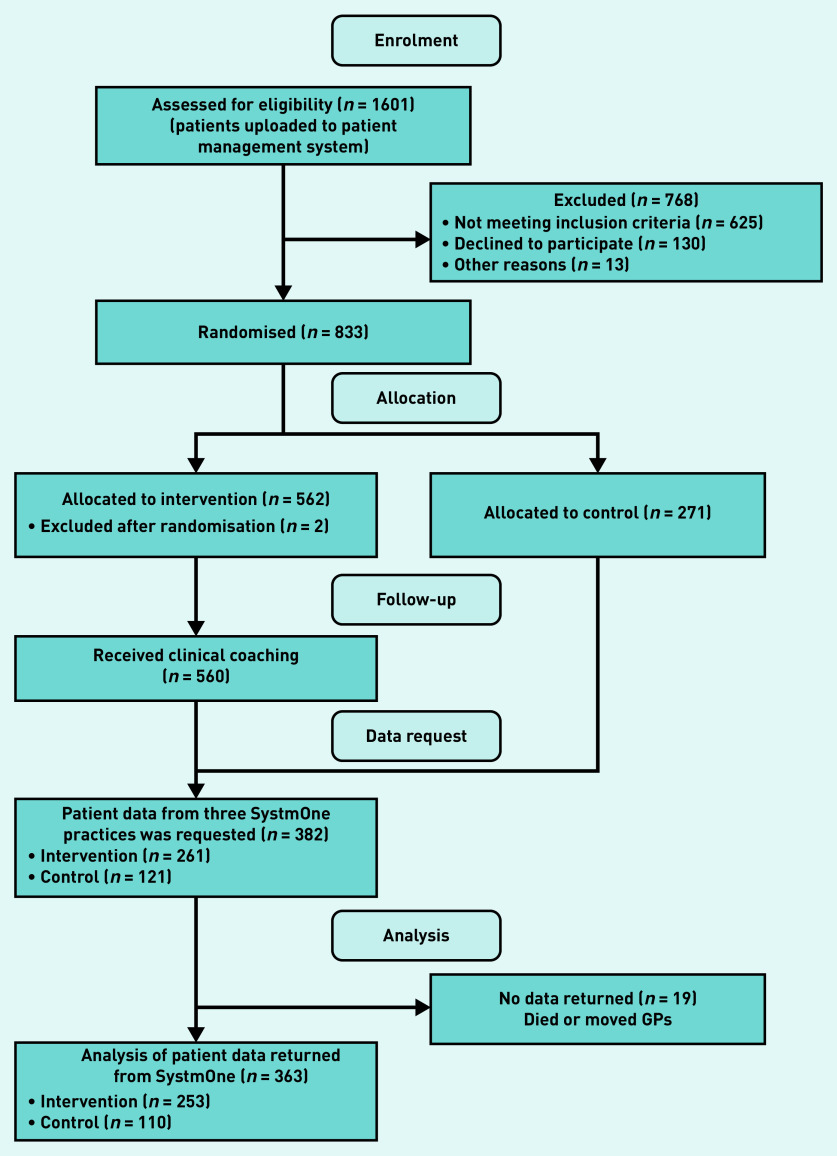
*CONSORT flow diagram.*

A total of 382 patients were recruited between 1 August 2015 and 1 November 2018. There were 19 patients who were excluded because of death or moving GP practice as permission to access their patient data was unavailable (Figure 1). There were 363 patients included in the analyses, 253 patients in the intervention arm and 110 patients in the control arm. All included patients had data for the 2-year follow-up post-randomisation. Both arms were comparable with regards to age, sex, and socioeconomic status (Table 1). The mean age was 71 years in the intervention arm and 72 years in the control arm. In total, 24% of the patients in the intervention arm were aged ≥80 years compared with 33% in the control arm. Half of the patients in both arms were from the most deprived socioeconomic group.

**Table 1.** Baseline characteristics of the study population (*n* = 363)

**Characteristic**	**Intervention group, % (*n* = 253, 69.7%)**	**Control group, % (*n* = 110, 30.3%)**	***P*-value^a^**
**Sex, female, *n* (%)**	127 (50.2)	65 (59.1)	0.12

**Age at randomisation, years, *n* (%)**			0.28
<45	8 (3.2)	3 (2.7)	
45–64	48 (19.0)	23 (20.9)	
65–79	136 (53.8)	48 (43.6)	
≥80	61 (24.1)	36 (32.7)	

**Age, years, mean (SD)**	71.3 (11.8)	72.2 (11.3)	

**IMD quintile, *n* (%)^b^**			0.55
1 (least deprived)	10 (4.0)	9 (8.2)	
2	39 (15.5)	14 (12.7)	
3	35 (13.9)	14 (12.7)	
4	42 (16.7)	18 (16.4)	
5 (most deprived)	125 (49.8)	55 (50.0)	

**Study entry year, *n* (%)**			0.74
2015	68 (26.9)	27 (24.5)	
2016	62 (24.5)	28 (25.5)	
2017	46 (18.2)	25 (22.7)	
2018	77 (30.4)	30 (27.3)	

a P*-value from χ^2^ tests.*

b *Data are only available for 251 patients in the intervention group. IMD = Index of Multiple Deprivation. SD = standard deviation.*

### Primary care use by type

#### Patient contacts

There was no significant difference in the mean annual rate of all primary care events between the intervention and control arms; with 46 events per person per year in both (IRR 1.00, 95% CI = 0.98 to 1.03, *P* = 0.70, Table 2).

**Table 2.** Annual rate of all primary care events in patients who received the Health Navigator intervention (*n* = 253) and the control group (*n* = 110) (calculated as total number of events per person per year)

**Event**	**Rate (95% CI)**	**IRR (95% CI)**	***P*-value^a^**
**All events**			
Control	46.08 (45.19 to 46.98)	1	0.70
Intervention	46.29 (45.70 to 46.89)	1.00 (0.98 to 1.03)	

**Face-to-face**			
Control	25.04 (24.36 to 25.71)	1	0.31
Intervention	24.63 (24.20 to 25.07)	0.98 (0.95 to 1.02)	

**Telephone**			
Control	5.92 (5.61 to 6.25)	1	0.64
Intervention	6.01 (5.80 to 6.23)	1.02 (0.95 to 1.08)	

**Letter**			
Control	12.95 (12.48 to 13.48)	1	0.01
Intervention	13.71 (13.39 to 14.04)	1.06 (1.01 to 1.11)	

**Community referral**			
Control	0.91 (0.79 to 1.04)	1	0.67
Intervention	0.94 (0.86 to 1.03)	1.04 (0.88 to 1.22)	

**Secondary care referral**			
Control	1.21 (1.08 to 1.37)	1	<0.001
Intervention	0.90 (0.82 to 0.99)	0.74 (0.64 to 0.86)	

a P*-value from Poisson regression model comparing rate in intervention group and control groups. CI = confidence interval. IRR = incident rate ratio comparing rate in intervention group with control group.*

When observing specific types of primary care use, there was a 6% increase in the mean annual rate of letters sent to patients in the intervention arm compared with the control arm (IRR 1.06, 95% CI = 1.01 to 1.11, *P* = 0.01). No difference was observed in the rate of face-to-face consultations or telephone contacts between the two arms (IRR 0.98, 95% CI = 0.95 to 1.02, *P* = 0.31; IRR 1.02, 95% CI = 0.95 to 1.08, *P* = 0.64, respectively, Table 2).

#### Patient referrals

A 26% decrease was observed in the annual rate of secondary care referrals in the intervention arm compared with the control arm (IRR 0.74, 95% CI = 0.64 to 0.86, *P*<0.001). No difference was observed in community referral rates between the two arms (IRR 1.04, 95% CI = 0.88 to 1.22, *P* = 0.67, Table 2).

### Primary care use by patient demographics

Patients aged ≥80 years in the intervention group had a 33% increase in the mean annual rate of primary care events compared with the control arm (IRR 1.33, 95% CI = 1.28 to 1.40, *P*<0.001), with a corresponding 10% decrease in events in patients aged <80 years (IRR 0.90, 95% CI = 0.87 to 0.92, *P*<0.001, Table 3). There was a 7% reduction in the mean annual rate of primary care events in males and a 7% increase in females in the intervention arm compared with the control arm (IRR 0.93, 95% CI = 0.89 to 0.96, *P*<0.001; IRR 1.07, 95% CI = 1.04 to 1.11, *P*<0.001, respectively). In the most affluent group, although numbers were small, a 49% increase in the rate of primary care events was found in the intervention arm compared with the control arm (IRR 1.49, 95% CI = 1.36 to 1.63, *P*<0.001).

**Table 3.** Annual rate of all primary care events in patients who had the Health Navigator intervention (*n* = 253) and the control group (*n* = 110) (calculated as total number of events per person per year) by sex, age, and socioeconomic status

**Characteristic**	**Intervention group, rate (95% CI)**	**Control group, rate (95% CI)**	**IRR (95% CI)**	***P*-value^a^**
**Sex**				
Male	44.66 (44.83 to 45.49)	48.23 (46.82 to 49.69)	0.93 (0.89 to 0.96)	<0.001
Female	47.92 (47.07 to 48.77)	44.59 (43.45 to 45.74)	1.07 (1.04 to 1.11)	<0.001

**Age at inclusion, years**				
<80	44.44 (43.78 to 45.12)	49.50 (48.38 to 50.65)	0.90 (0.87 to 0.92)	<0.001
≥80	52.11 (50.85 to 53.41)	39.04 (37.62 to 40.51)	1.33 (1.28 to 1.40)	<0.001

**IMD quintile**				
1 (least deprived)	60.81 (57.49 to 64.32)	40.92 (38.07 to 43.98)	1.49 (1.36 to 1.63)	<0.001
2	48.80 (7.28 to 50.38)	48.32 (45.82 to 50.97)	1.00 (0.95 to 1.07)	0.76
3	41.14 (39.67 to 42.67)	51.72 (49.12 to 54.45)	0.80 (0.75 to 0.85)	<0.001
4	47.55 (46.10 to 49.05)	48.13 (45.92 to 50.45)	0.99 (0.93 to 1.05)	0.67
5 (most deprived)	45.81 (44.98 to 46.66)	44.24 (43.02 to 45.50)	1.04 (1.00 to 1.07)	0.04

a P*-value from Poisson regression model comparing rate in intervention group and control groups. CI = confidence interval. IMD = Index of Multiple Deprivation. IRR= incident rate ratio comparing rate in intervention group with control group.*

### Primary care use over time

#### All primary care events

**Figure 2.**
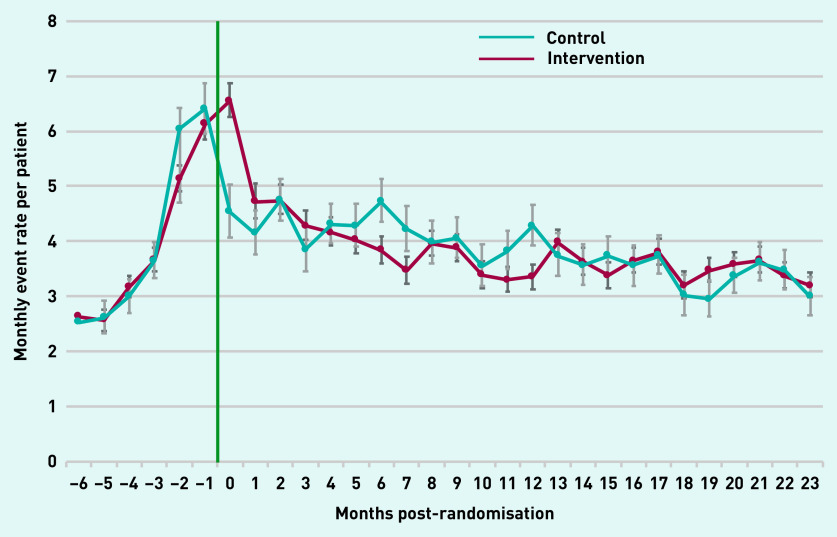
***Monthly rate of all primary care events over time post-randomisation compared with control group.*** *^a^* *^a^* ***Green line indicates start of the Health Navigator intervention.***

A rapid increase in primary care use during the 6 months preceding the intervention was observed in both arms. In the intervention arm, the monthly rate of primary care events rose from a baseline of 2.5 events per patient at −6 months to 6.2 events per patient at the time of the intervention, with a similar trend in the control arm (Figure 2). Following randomisation, in both arms an initial rapid decline in the monthly rate of primary care events was found in the first 2 months, followed by a slower downward trend towards the baseline rate.

#### Primary care events by type

**Figure 3.**
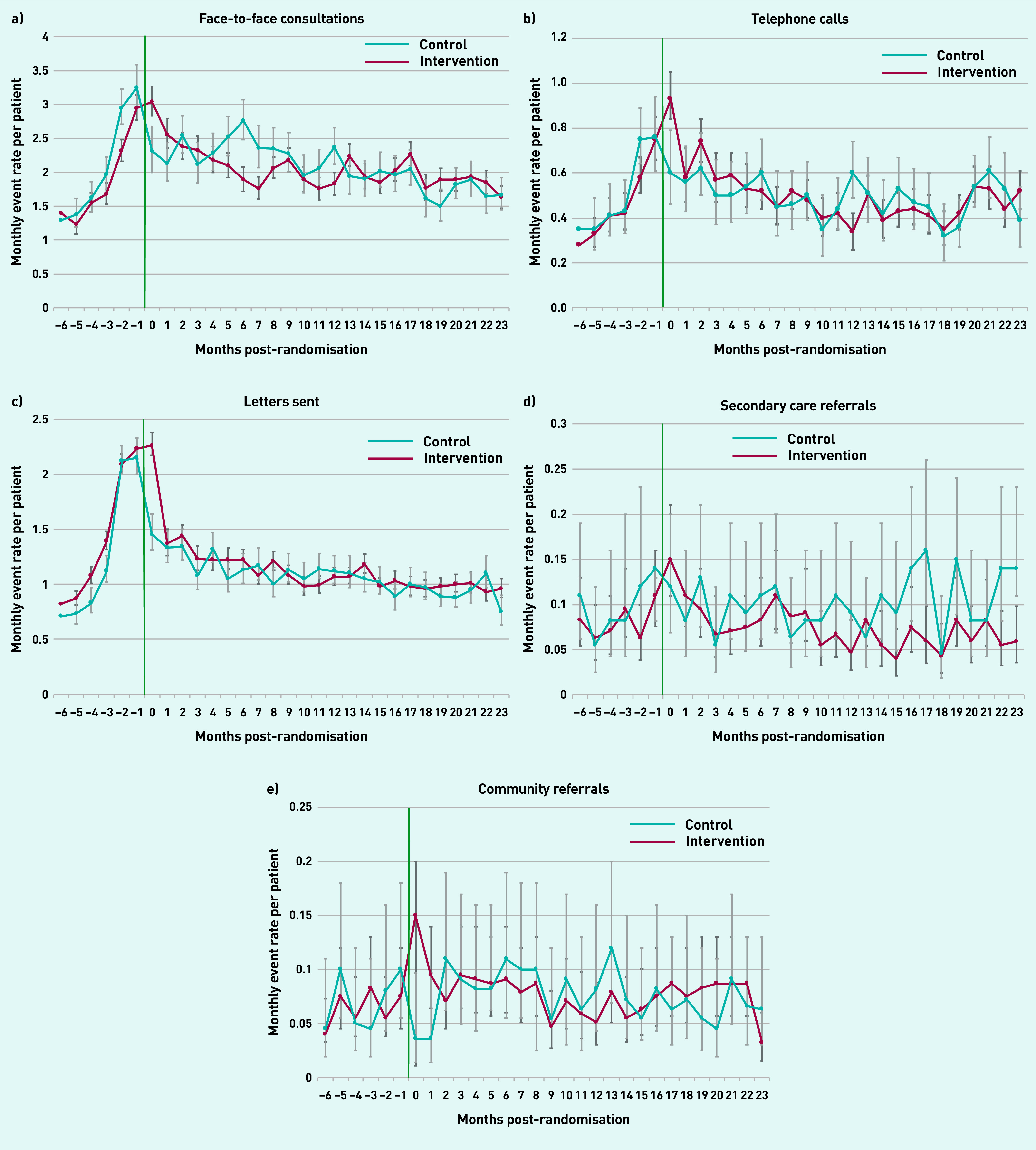
***Monthly rate of different primary care events over time post-randomisation compared with control group. a) Face-to-face consultations; b) telephone calls; c) letters sent; d) secondary care referrals; and e) community referrals.*** *^a^* *^a^* ***Green line indicates start of the Health Navigator intervention.***

Similar trends were observed for use of the five different types of primary care events (Figure 3), with comparable monthly rates over time observed in the intervention and control arms. In the intervention arm, face-to-face consultations dropped from a monthly rate of 3.0 in the month immediately post-randomisation to a rate of 1.6 at 2 years post-randomisation; telephone consultations dropped from 0.8 per month to 0.5 at 2 years post-randomisation; and letters dropped from 2.3 per month to 0.9 at 2 years, with comparable rates seen in the control arm. When examining referral rates over time, both community and secondary care referral rates were stable for the whole study period in both arms.

### Primary care events by patient demographics

**Figure 4.**
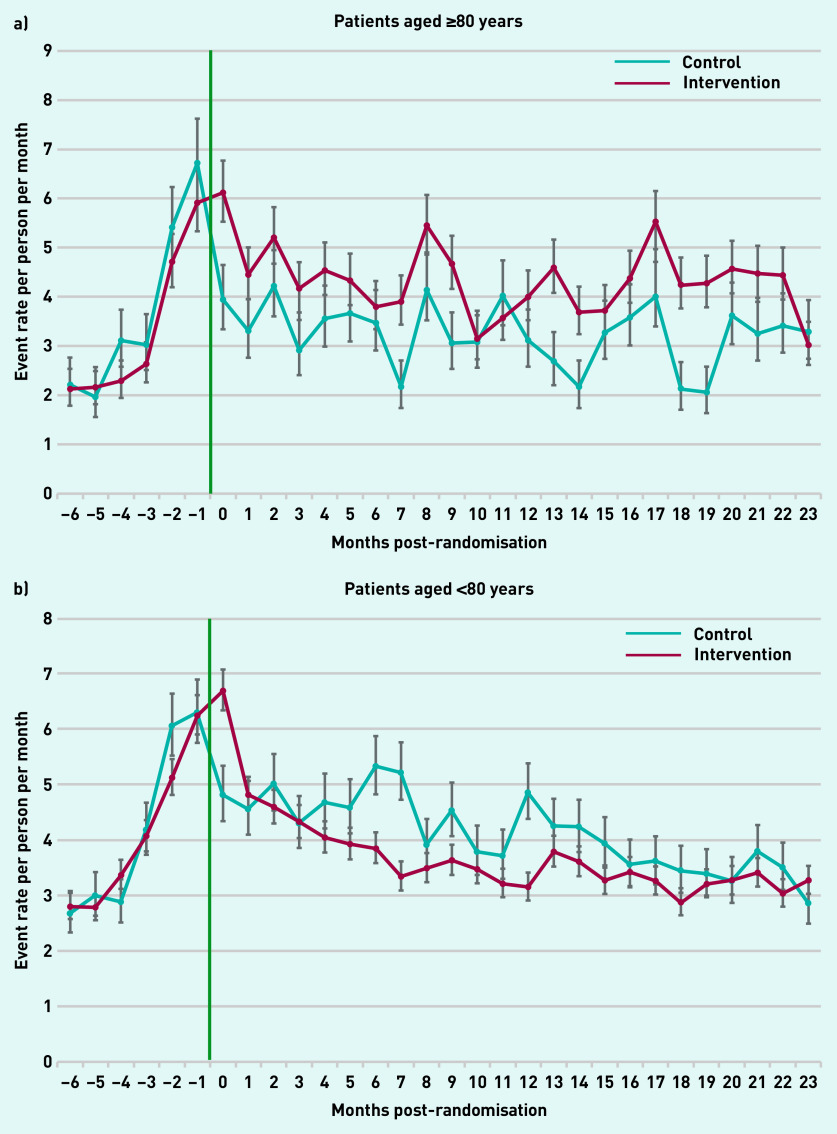
***Monthly rate of all primary care events over time post-randomisation by age group. a) Patients aged ≥80 years; and b) patients aged <80 years.*** *^a^* *^a^* ***Green line indicates start of the Health Navigator intervention.***

Patients aged ≥80 years in the intervention arm had higher monthly rates of primary care use for the 2 years following the intervention compared with the control group in all but 2 months (Figure 4). Correspondingly, patients aged <80 years in the intervention arm had lower monthly rates in 21 of 24 months compared with controls. Primary care use over time was comparable for males and females in both arms (Supplementary Figure S3).

## DISCUSSION

### Summary

This case-management intervention for frequent users of ED services had no overall impact on primary care use when examining all primary care events across the study population. There was a statistically significant decrease in secondary care referrals and an increase in letters sent to patients in the intervention arm. The intervention has differing effects on primary care use in specific patient groups, with an increase in use in those aged ≥80 years and female, and a decrease in those aged <80 years and male. A steep increase in primary care use was observed during the 6 months before the intervention indicating that data from GP EHRs could be used to potentially enhance existing risk-prediction models to identify patients at increased risk of ED attendance earlier.

### Strengths and limitations

The strengths of the study include the use of patient-level GP data from EHRs and the RCT study design. The granular nature of these data enabled comprehensive evaluation of the impact on specific aspects of primary care use. The RCT design allowed comparison with a control group, with randomisation and intention-to-treat analysis minimising bias and ensuring comparability between the two arms. Additionally, the 2-year follow-up period enabled examination of trends over time and to establish if any effects were sustained. The large sample size allowed examination of effects by patient demographic and type of primary care use.

The study used routinely collected data. Practices may vary in the accuracy and completeness of their recording and missing data may mean that the figures for primary care use are underestimates; however, this will affect both arms equally. Participating patients in both arms may be susceptible to the Hawthorne effect;^26^ however, this is likely to be minimal as data are remotely extracted from EHRs without direct patient involvement or observation.

The population of the Vale of York may not be representative of the UK population; patients identified by the artificial intelligence algorithm in this study may therefore differ from those identified in other regions of the UK. This has implications for the generalisability of the study findings across other UK regions and further work is needed to confirm these findings in a larger population. Additionally, exclusion criteria were applied to select patients most likely to be able to engage with telehealth interventions; therefore, findings will only be generalisable to similarly selected patient groups.

The study identified and includes patients who were using healthcare services at an increased rate. The subsequent rate of healthcare use may include an element of regression to the mean. Finally, the authors recognise there were changes in healthcare delivery and patient health-seeking behaviour in response to the COVID-19 pandemic. Sensitivity analysis found excluding follow-up from 1 March 2020 did not have an impact on the findings of this study. Future research of primary care use should explore use of video and e-consults.

### Comparison with existing literature

Previous studies have demonstrated that case-management interventions to reduce ED admissions are effective;^17^^–^^19^^,^^27^^–^^29^ however, their impact on other services has not been explored.

Studies of case-management interventions for purposes other than reducing ED attendances have examined some of the impact on primary care use. A case-management intervention for patients with chronic disease was found to have led to a 5% reduction in short-term primary care use; however, this was based on quasi-experimental qualitative data from patient questionnaire estimations of usage rather than quantitative data.^30^ Conversely, a meta-analysis on case-management interventions for patients at risk of admission to hospital did not find any significant impact on use of primary care.^31^ However, that study only examined a composite outcome of GP visits combined with home care, social worker, and nursing visits. A systematic review of case-management services to integrate care between healthcare services found that two UK studies observed a reduction in GP appointments and one found no difference, concluding further work was needed in this area.^32^

The current study, to the authors’ knowledge, for the first time quantifies the impact on primary care of a case-management intervention designed to reduce ED attendances. It expands on previous studies by exploring the impact on different types of primary care use, the effect in different patient groups, and the effect over time for 2 years following the introduction of the intervention. Future studies should examine the effects of the intervention on wider health services, including further community services, and examine whether there are sustained effects over a longer time period.

## Implications for practice

This study has shown that an intervention that reduces ED attendances does not create additional workload in primary care. In addition, the decrease in secondary care referrals may reduce hospital workload. This may be because of patients having their conditions managed effectively in the community, supported by health coaches, without the need for onward referral, thereby freeing capacity in secondary care. A previous study has shown that continuity of care is associated with reduced secondary care usage.^12^ The continuity provided by the health coaches in this study may contribute to lower use of other services.

The intervention is comprehensive but could be economically scaled up. Each nurse has an active caseload of 40–50 patients and supports 120 patients per year (personal communication, Nurse Specialist from Health Navigator, 2022). As high-risk patients for unplanned care are a small proportion of all patients, a large workforce would not be required to expand the intervention into larger healthcare systems. Given the reduction in secondary care referrals, this may represent a good use of resources.

To further understand the implications of these results, first what a ‘good’ outcome is needs to be considered. This study found that patients aged ≥80 years had increased primary care usage, with those aged <80 years experiencing a reduction. The majority of patients with complex medical needs are aged >80 years.^33^ A possible inference from these findings is that this group experienced prior unmet clinical needs, challenges accessing their GP, or a lack of knowledge of available services. The intervention potentially facilitates and resolves these issues, enabling these patients to have increased appropriate contact with their GP. This may be considered an ‘ideal’ outcome as care of chronic disease is optimised in primary care.

The corresponding reduction in primary care contact in patients aged <80 years, where the collective burden of chronic disease is lower, may indicate that the intervention supports these patients effectively without the need for their GP’s involvement or secondary care referral. Although this could potentially reduce opportunities for health promotion, this is mitigated by routine monitoring and follow-up, practice reminder systems, and initiatives, such as the Quality and Outcomes Framework.^34^

The marked increase in primary care events during the 6 months before the intervention has implications for future risk-prediction modelling. The existing algorithm exclusively uses hospital data to identify patients at high risk of accessing emergency care. Incorporating routinely collected primary care data from existing large, nationwide datasets into risk-prediction models could improve the current model’s accuracy in detecting high-risk patients, identify patients as high risk sooner before accessing unscheduled care, and enable prediction of other outcomes, such as increased primary care use to enable earlier intervention.

The implications of this study can be framed in a broader outlook when planning an integrated health service and commissioning further services in the future; interventions that seek to encourage patients away from an existing pathway may have an impact elsewhere in the system. However, patient needs may be more appropriately and efficiently met in different settings, which could reduce overall utilisation of healthcare resources. This study demonstrates that a case-management intervention, designed to reduce ED attendances, does not have an impact on overall primary care use and may redistribute services to those with greater clinical need.

## Data

All data requests should be directed to Health Navigator.
